# MCPerm: A Monte Carlo Permutation Method for Accurately Correcting the Multiple Testing in a Meta-Analysis of Genetic Association Studies

**DOI:** 10.1371/journal.pone.0089212

**Published:** 2014-02-21

**Authors:** Yongshuai Jiang, Lanying Zhang, Fanwu Kong, Mingming Zhang, Hongchao Lv, Guiyou Liu, Mingzhi Liao, Rennan Feng, Jin Li, Ruijie Zhang

**Affiliations:** 1 College of Bioinformatics Science and Technology, Harbin Medical University, Harbin, China; 2 Department of Nephrology, The Second Affiliated Hospital, Harbin Medical University, Harbin, China; 3 Genome Analysis Laboratory, Tianjin Institute of Industrial Biotechnology, Chinese Academy of Sciences, Tianjin, China; 4 College of Life Science, Northwest A&F University, Yangling, Shaanxi, China; 5 Department of Nutrition and Food Hygiene, School of Public Health, Harbin Medical University, Harbin, China; University of East Piedmont, Italy

## Abstract

Traditional permutation (TradPerm) tests are usually considered the gold standard for multiple testing corrections. However, they can be difficult to complete for the meta-analyses of genetic association studies based on multiple single nucleotide polymorphism loci as they depend on individual-level genotype and phenotype data to perform random shuffles, which are not easy to obtain. Most meta-analyses have therefore been performed using summary statistics from previously published studies. To carry out a permutation using only genotype counts without changing the size of the TradPerm *P*-value, we developed a Monte Carlo permutation (MCPerm) method. First, for each study included in the meta-analysis, we used a two-step hypergeometric distribution to generate a random number of genotypes in cases and controls. We then carried out a meta-analysis using these random genotype data. Finally, we obtained the corrected permutation *P*-value of the meta-analysis by repeating the entire process *N* times. We used five real datasets and five simulation datasets to evaluate the MCPerm method and our results showed the following: (1) MCPerm requires only the summary statistics of the genotype, without the need for individual-level data; (2) Genotype counts generated by our two-step hypergeometric distributions had the same distributions as genotype counts generated by shuffling; (3) MCPerm had almost exactly the same permutation *P*-values as TradPerm (*r* = 0.999; *P*<2.2e-16); (4) The calculation speed of MCPerm is much faster than that of TradPerm. In summary, MCPerm appears to be a viable alternative to TradPerm, and we have developed it as a freely available R package at CRAN: http://cran.r-project.org/web/packages/MCPerm/index.html.

## Introduction

Meta-analysis is an important method that can improve statistical power by combining the results of multiple previously published studies [1], [2]. Every year, thousands of meta-analyses of genetic association studies are published. Some focus on only one single nucleotide polymorphic (SNP) locus [3]–[5], while others consider multiple SNP loci [6]–[9]. For those meta-analyses based on multiple loci, multiple testing is a persistent problem. Many techniques have been devised to correct multiple hypotheses, such as the Bonferroni and Sidak corrections [10], false discovery rate [11], and permutation [12]. Although these methods are highly successful, the traditional permutation (TradPerm) method is still widely considered the gold standard for accurately correcting for multiple testing [12], [13].

TradPerm is a type of non-parametric method in which the null distribution of the test statistic is estimated by shuffling uniform phenotype labels of cases and controls [13], [14]. However, it relies on the original SNP genotype and phenotype data (individual-level data) to perform a large number of random shuffles [15]. For the meta-analyses of genetic association studies, individual-level data are difficult to obtain for several reasons including research project privacy. Compared with raw SNP genotype data, count-based summary statistics of genotype data are more readily available, so most meta-analyses of genetic associations have been performed using summary statistics from previously published studies. However, for researchers of meta-analyses, it can be challenging to perform the permutation test using only the summary statistics of the genotype when similarly high standards to TradPerm are expected.

Here, we developed a Monte Carlo permutation (MCPerm) method for use in the permutation test. MCPerm employs a two-step hypergeometric distribution to generate random genotype counts in cases and controls. We used these random genotype counts to construct the background distribution of meta-analysis *P*-values and to complete the permutation correction. Finally, MCPerm was evaluated using five sets of real data and five sets of simulation data.

## Methods

### Data

The five real data sets used to evaluate the MCPerm method were the AlzGene database (Field Synopsis of Genetic Association Studies in Alzheimer disease, www.alzgene.org), SZGene database (Field Synopsis of Genetic Association Studies in Schizophrenia, www.szgene.org), PDGene database (Field Synopsis of Genetic Association Studies in Parkinson’s Disease, www.pdgene.org), MSGene database (Field Synopsis of Genetic Association Studies in Multiple Sclerosis, www.msgene.org), and AlsGene database (Field Synopsis of Genetic Association Studies in Amyotrophic Lateral Sclerosis, www.alsgene.org) [6]–[9]. We used PERLscript to extract all records from the five databases. The following criteria were used to filter the data: (1) all records must have a case-control design; (2) all records must have SNP genotype counts for both cases and controls; (3) the minor allele frequency must be greater than 0.01 for single records; (4) the *P*-value of the Hardy-Weinberg equilibrium test in the control group must be greater than 0.001 for single records; (5) each SNP must be reported by at least four studies (which will be used in the meta-analysis). We also excluded family-based studies.

Each record included the following information: study ID, the first author’s name, the year of publication, ethnicity, SNP names, SNP alleles, SNP genotypes, and genotype counts for cases and controls. A total of 850 SNP loci were obtained for the meta-analysis, and each locus had at least four records. These included 287 SNP loci from the AlzGene database (a total of 2,408 records), 273 from the SZGene database (a total of 2,179 records), 182 from the PDGene database (a total of 1,625 records), 88 from the MSGene database (a total of 604 records), and 20 from the AlsGene database (a total of 161 records).

As TradPerm relies on individual-level data, we also constructed five simulation data sets. We generated the individual’s genotype (*AA*, *Aa*, or *aa*) for each record based on the genotype count in cases and controls. By shuffling these simulated data, we constructed the random background and completed the TradPerm correction.

### Meta-analysis

For ease of understanding, we will first describe the meta-analysis method. Suppose there are two alleles, *A* (*A* is the risk allele) and *a* at SNP locus A. Three possible genotypes are denoted by *AA*, *Aa*, and *aa*. Suppose also that a meta-analysis includes 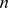 previously published studies. Without loss of generality, we mainly considered the allele model in the present study (the effect of the *A* allele vs. the *a* allele); however, we also provided some findings about the dominant and recessive models, and used 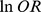 to measure the effect size. To carry out a meta-analysis, we first tested for heterogeneity between studies. The commonly used indicators are Cochran’s *Q*-statistics and *I* ^2^ [16], [17]. In this study, we used Cochran’s *Q*-statistics to evaluate heterogeneity. The null hypothesis was that the *n* studies have the same effect, and the significance level was 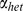. It should be noted that the multiple testing problem ought to be considered for the heterogeneity test in the meta-analyses of multiple loci, although few researchers do so. We provided a permutation correction method for the heterogeneity test, and, after testing for heterogeneity, we combined the effect sizes and evaluated the statistical association between genotype and phenotype. If heterogeneity existed (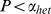), we used a random effects model (assuming that each study has a specific effect size) to combine the effect sizes. If heterogeneity did not exist (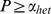), we used a fixed effects model (assuming that all studies share a common effect size) to combine the effect sizes [18].

### TradPerm

For multiple SNP loci, the problem of multiple testing correction should be considered, so we describe below the TradPerm method for multiple testing corrections.

**Figure 1:**
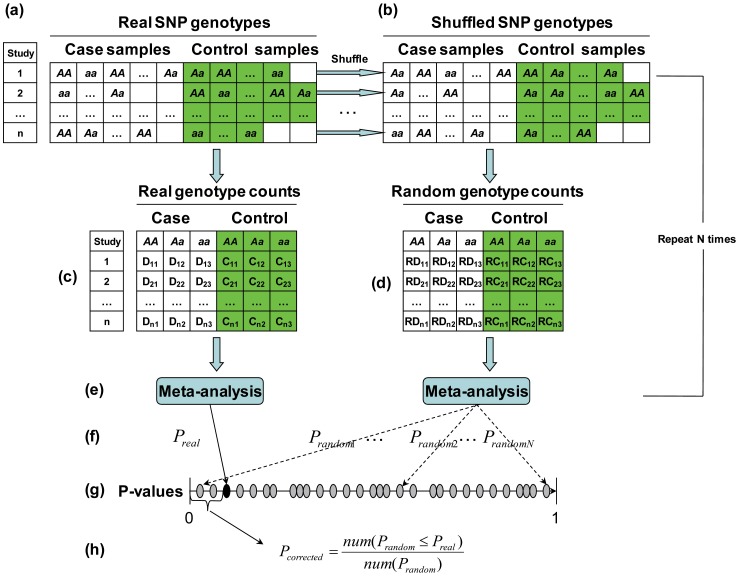
TradPerm method. (a) Real individual-level genotype. (b) Shuffled individual-level genotype. (c) Genotype count from real SNP genotype. (d) Genotype count from random SNP genotype. (e) Completing meta-analysis. (f) Calculating *P*-values of meta-analysis (real *P*-values and *N* random *P*-values). (g) Ranking all *P*-values. (h) Calculating the corrected *P*-value.

Suppose that we have obtained the original SNP genotype and phenotype data (individual-level data) from *n* previously published studies (Figure 1a). Each study has a different sample size. For each study, we can count the number of each of the three genotypes (*AA*, *Aa*, and *aa*) in the cases and controls (Figure 1c). Using these genotype counts, we can perform a meta-analysis and obtain a meta-analysis *P*-value (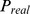), then carry out a TradPerm procedure to correct the.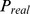.

The principle of TradPerm is if there is no association between genotypes and phenotypes, then any of the individual phenotype labels may be associated with any one of the genotypes. Therefore, the background distribution of the meta-analysis *P*-value can be generated by randomly shuffling the phenotype labels of cases and controls. Specifically, for each study included in the meta-analysis, we shuffled the phenotype labels (Figure 1b) and re-counted the number of the three genotypes in cases and controls (Figure 1d). Using these random genotype counts, we performed a meta-analysis (Figure 1e) and obtained a random meta-analysis *P*-value (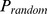). The entire process was repeated *N* times to obtain 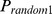, 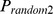,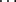, 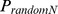 (Figure 1f). If there is a significant association between genotypes and phenotypes, the 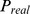 calculated using the real data will appear small relative to the distribution of the *P*-values obtained under permutation (Figure 1g). In other words, if the association does not exist, the 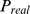 for real data is unlikely to be obtained. The TradPerm *P*-value (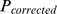) under the null hypothesis can be calculated as the proportion of *P*-values under permutation that are less than or equal to 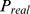 (Figure 1h). 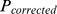 is defined as follows: [19].
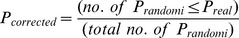
where 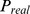 is the real meta-analysis *P*-value, and 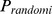 is the *P*-value generated by permutation. Similarly, the *P*-value of the heterogeneity test can be corrected by shuffling the phenotype labels of cases and controls.

### MCPerm

As described above, TradPerm relies on individual-level data which are very difficult to obtain. Therefore, our MCPerm method only uses count-based summary genotype data. MCPerm employs a two-step hypergeometric distribution to generate the random genotype counts in cases and controls.

#### Step 1: Simulate genotype count of AA in cases

First, we will consider one study in a meta-analysis. We suppose that there are a total of 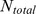 samples, including 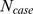 cases and 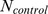
controls in this study (Table 1). Consider locus A, where the counts of the three genotypes in cases and controls were denoted as: 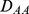 for *AA* in cases, 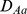 for *Aa* in cases, 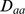 for *aa* in cases, 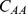 for *AA* in controls, 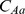 for *Aa* in controls, and 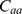 for *aa* in controls. The total number of counts of the three genotypes in all samples was denoted as: 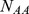 for *AA*, 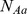 for *Aa*, and 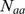 for *aa.* Next, we generated the genotype counts, 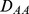, by stochastic simulation.

**Table 1:** The 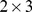 genotypic table.

	*AA*	*Aa*	*Aa*	Total
Case	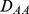	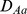	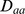	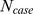
Control	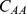	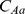	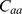	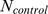
Total	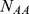	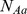	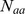	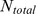

First, we will introduce the hypergeometric distribution in statistics. Suppose an urn contains 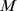 balls of which 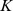 are black and 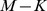 are white. Consider an experiment in which 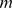 balls are drawn without replacement from the run. The number of black balls in the sample of 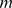 obeys a hypergeometric distribution: 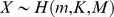.

Now we will consider the entire TradPerm process. There are a total of 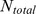 samples of which 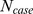 are cases and 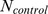 are controls (where 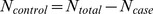). When we randomly shuffle the phenotype labels of cases and controls, the count of genotype *AA* in all samples is unchanged (that is, 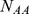 is a constant). This is equivalent to randomly drawing 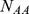 samples without replacement. Therefore, the count of genotype *AA* in case samples obeys a hypergeometric distribution 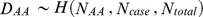; i.e.,
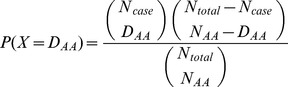
Finally, we used the hypergeometric random number to simulate the count of genotype *AA* in case samples (that is, 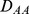). Kachitvichyanukul’s method [20] was used to generate the hypergeometric random number. This can be achieved by the function “rhyper” in R language. Because 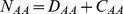 is a constant when we randomly shuffle, we can deduce that 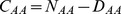 (where 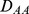 is a hypergeometric random number generated by Kachitvichyanukul’s method).

#### Step 2: Simulate genotype count of Aa in cases

At this stage, we have fixed the counts of genotype *AA* in case and control samples. As randomly shuffling is equivalent to randomly sampling without replacement, the remaining count of samples is 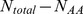. When we randomly shuffle the phenotype labels, the count of genotype *Aa* remains unchanged (that is, 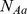 is a constant). This is equivalent to randomly drawing 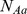 samples from the 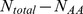 remaining samples without replacement. Therefore, the count of genotype *Aa* in case samples also obeys the hypergeometric distribution 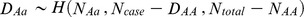; i.e.,
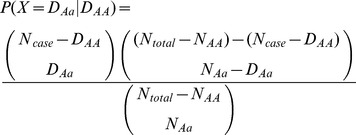
Therefore, we still employ Kachitvichyanukul’s method [20] to generate the hypergeometric random number. Because 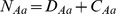 is a constant, we can deduce 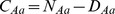 (where 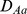 is a hypergeometric random number generated by Kachitvichyanukul’s method).

Finally, we can deduce 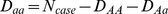 and 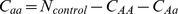. Table 2 summarizes the methods used to simulate the six genotype counts.

**Table 2:** Summary of simulated methods of six genotype counts.

genotypes	counts	generationmethods
*AA* in case	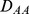	hypergeometric random number generated in step1
*Aa* in case	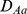	hypergeometric random number generated in step2
*aa* in case	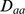	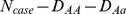
*AA* in control	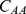	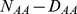
*Aa* in control	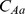	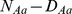
*aa* in control	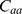	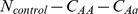
total	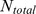	

#### MCPerm procedure

**Figure 2:**
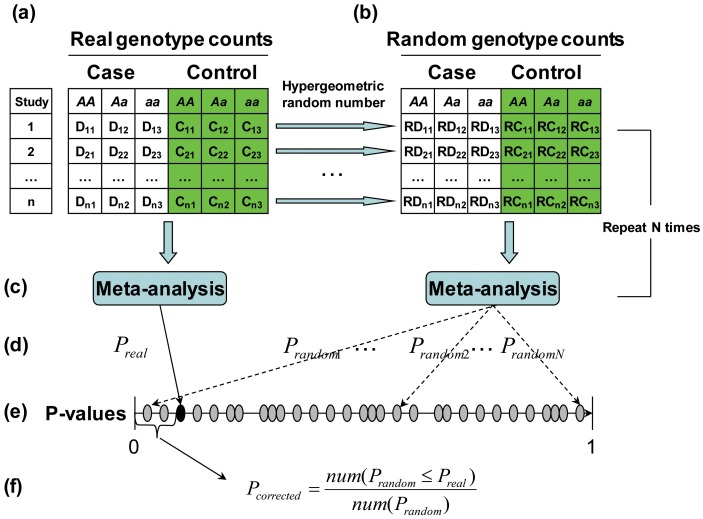
MCPerm method. (a) Genotype count collected from previous studies. (b) Genotype count generated by two-step hypergeometric distribution. (c) Completing meta-analysis. (d) Calculating *P*-values of meta-analysis (real *P*-values and *N* random *P*-values). (e) Ranking all *P*-values. (f) Calculating the corrected *P*-value.

The MCPerm *P*-value was calculated in the same way as for TradPerm. Specifically, for each study, we randomly generated genotype counts in cases and controls (Figure 2b). We then carried out a meta-analysis (Figure 2c) and obtained a random meta-analysis *P*-value (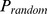). The entire process was repeated 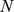 times, resulting in 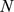 random P-values (Figures 2d and 2e). The MCPerm *P*-value (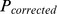) can also be calculated as the proportion of *P*-values under simulation that are less than or equal to 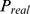
(Figure 2f). Similarly, the *P*-value of the heterogeneity test can be corrected by simulating the genotype counts of cases and controls.

## Results

As TradPerm is often treated as the gold standard for multiple testing corrections, we did not compare our MCPerm with other multiple testing corrections methods. Instead, we compared the consistency of genotype distributions and permutation *P*-values between TradPerm and MCPerm. Our aim was to illustrate that MCPerm does not change the size of TradPerm *P*-values, so is a useful alternative to TradPerm.

### Comparing Six Genotype Distributions between MCPerm and TradPerm

**Figure 3:**
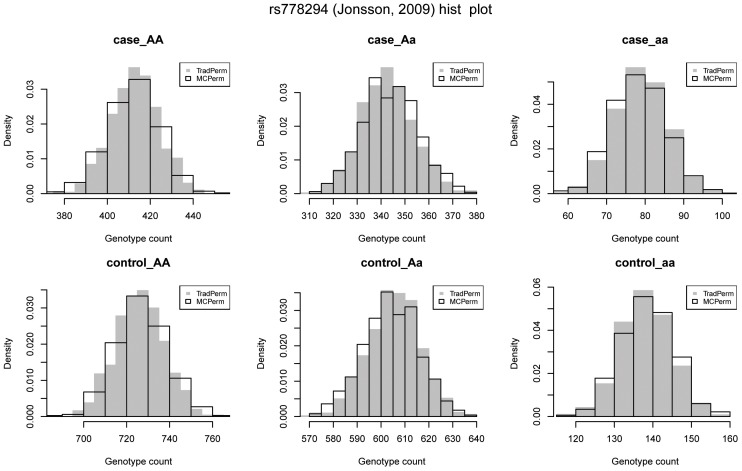
Distributions of the six genotype counts. *AA*, *Aa*, *aa* in cases, and *AA*, *Aa*, and *aa* in controls. Gray solid bars represent the distributions obtained under TradPerm, and black hollow bars represent the distributions generated by MCPerm. The distributions are the same between MCPerm and TradPerm.

As an example, we randomly selected one SNP, rs778294, from the SZGene database. There were a total of 12 studies about rs778294 in the SZ database, and we selected one ( [21]) to compare six genotype distributions between MCPerm and TradPerm. We generated the individual’s genotype for this study based on the genotype counts in cases and controls. First, we used TradPerm to shuffle the sample labels 1,000 times and calculated the counts of six genotypes for each shuffle. Then we used MCPerm to generate the counts of the six genotypes (again 1,000 times). Figure 3 shows the distributions of the genotype counts, and we can see that the six genotype counts under MCPerm had similar distributions to genotype counts under TradPerm. This can also be seen from the cumulative distribution curve (Figure S1).

We next used the Kolmogorov-Smirnov test (KS-test) to compare genotype distributions between MCPerm and TradPerm. The null hypothesis was that the genotype counts had the same distribution between MCPerm and TradPerm, and this null hypothesis was not rejected after performing the KS-test in all six tests. The six KS-test *P*-values were *P* = 0.069 for *AA* genotypes in cases, *P* = 0.148 for *Aa* genotypes in cases, *P* = 0.828 for *aa* genotypes in cases, *P* = 0.069 for *AA* genotypes in controls, *P* = 0.148 for *Aa* genotypes in controls, and *P* = 0.828 for *aa* genotypes in controls. Because 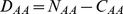, the *AA* genotypes in the cases had the same KS-test *P*-value as the *AA* genotypes in the controls (*P* = 0.999). Similarly, *Aa* and *aa* genotypes in the cases had the same KS-test *P*-values as the controls. This indicated that the six genotype counts under MCPerm had the same distributions as genotype counts under TradPerm.

**Figure 4:**
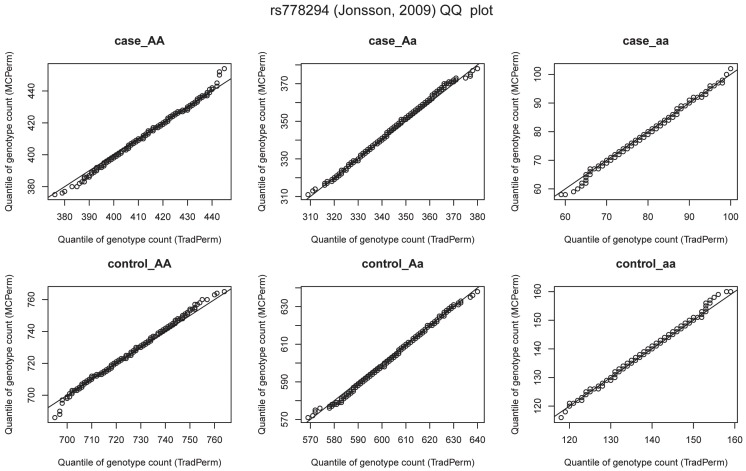
QQ-plots of six genotype counts. *AA*, *Aa*, and *aa* in cases, *AA*, *Aa*, and *aa* in controls.

We also used the quantile-quantile plot (QQ-plot) to evaluate the consistency of the genotype distributions between MCPerm and TradPerm. This plotted the quantiles of the genotype counts generated by MCPerm vs. the quantiles of the genotype counts calculated by TradPerm (from 1,000 permutations). Figure 4 shows the QQ-plots of the six genotypes which were all linear, indicating that the genotype distributions are the same between MCPerm and TradPerm.

In addition to genotype distributions, we also evaluated the distributions of the allele model (*A* allele vs. *a* allele, Figure S2), the dominant model (*AA+Aa* vs. *aa* genotypes, Figure S3), and the recessive model (*AA* vs. *Aa+aa* genotypes, Figure S4), and again observed the same distributions between MCPerm and TradPerm.

### Comparing Meta-analysis *P*-values between MCPerm and TradPerm

**Figure 5:**
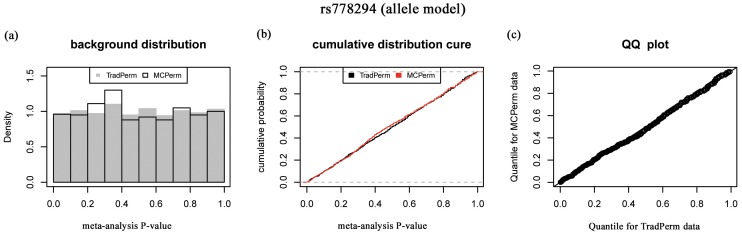
Comparison of meta-analysis *P*-values between MCPerm and TradPerm. (a) Probability density. (b) Cumulative distribution curve. (c) QQ-plot.

In this section, we will describe the consistency of meta-analysis *P*-values between TradPerm and MCPerm. For each of the 850 SNPs, we carried out 1,000 permutations and calculated the permutation *P*-values of the meta-analysis (allele model) using TradPerm and MCPerm. For SNP rs778294, the probability density (Figure 5A), cumulative distribution curve (Figure 5B), and QQ-plot (Figure 5C) showed that permutation *P*-values had the same distributions between MCPerm and TradPerm. The same conclusions were obtained for both the dominant model and recessive model (Figures S5 and S6), and for the other 849 SNP loci. The comparison results for all 850 loci are shown in Supporting Information S1, which can be downloaded at http://www.bioapp.org/research/MCPerm/index.html.

**Figure 6:**
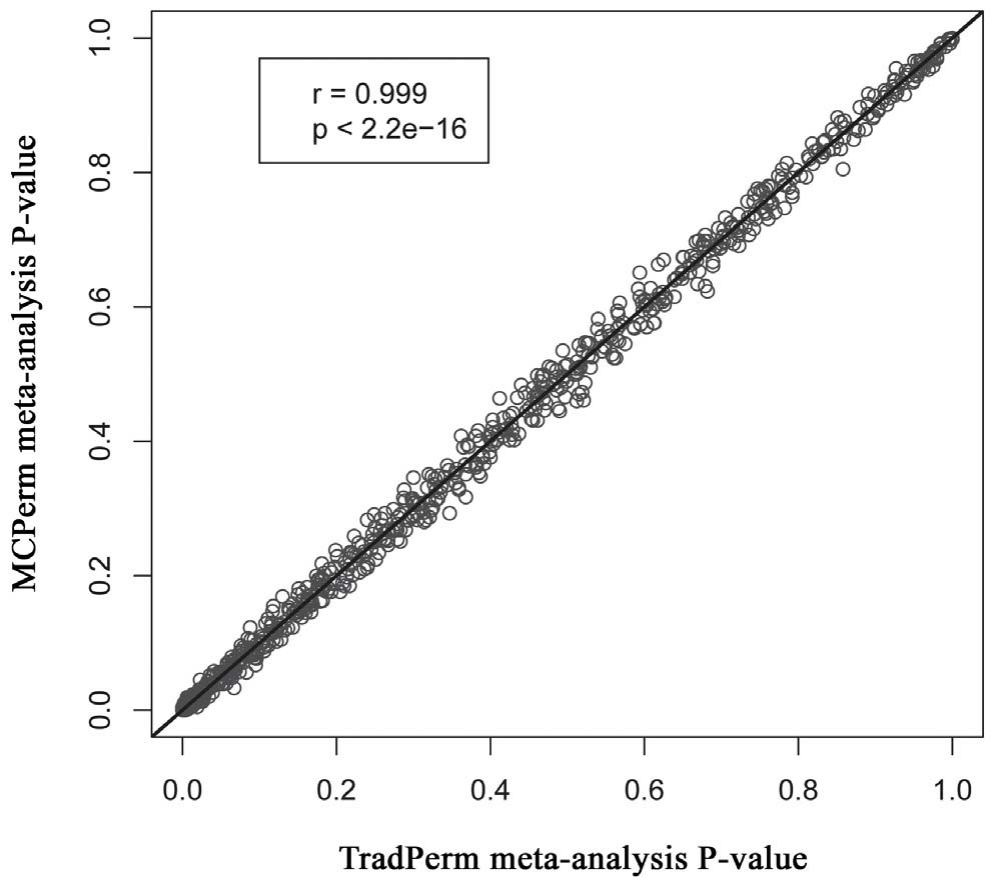
Scatter plots of 850 MCPerm vs. 850 TradPerm meta-analysis *P*-values. The MCPerm *P*-values are highly consistent with TradPerm *P*-values (*r = *0.999; *P*<2.2e-16).

We next plotted a scatter plot of 850 MCPerm meta-analysis *P*-values against 850 TradPerm meta-analysis *P*-values (Figure 6). This revealed a highly linear relationship between MCPerm *P*-values and TradPerm *P*-values. We also calculated the Pearson’s correlation coefficient of the correlation between MCPerm *P*-values and TradPerm *P*-values (*r = *0.999), and the correlation test *P*-value was <2.2e-16. These findings indicate that the MCPerm *P*-values are highly consistent with TradPerm *P*-values. The same conclusions were obtained for both the dominant model and recessive model.

### Comparing Heterogeneity Test *P*-values between MCPerm and TradPerm

For the 850 SNPs, we compared the consistency of heterogeneity test *P*-values. For SNP rs778294, the probability density, cumulative distribution curve, and QQ-plot results revealed the same distributions of heterogeneity test *P*-values between MCPerm and TradPerm (Figures S7, S8, and S9). The comparison results for all 850 loci are shown in Supporting Information S1. The correlation test (*r* = 0.999, *P*-value <2.2e-16) also showed a high consistency of heterogeneity test *P*-values between MCPerm and TradPerm (Figure S10).

### Estimating the Variance of lnOR and a Permutation Box Plot

In general, for a single study, the variance of 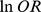
(in an allele model, dominant model, or recessive model) is estimated using the following formula: 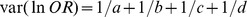, where *a*, *b*, *c*, and *d* represent the observed counts in fourfold tables [22]. The variance of 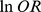
is used as the weight of a single study when multiple studies are combined in a meta-analysis. Obviously, different observed counts cause different estimations of the variance of 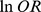, and over-reliance on the original observed counts will affect the stability of the results. An advantage of MCPerm is that we can simulate the background of 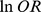 by a two-step hypergeometric random number. This enables us to directly calculate the variance of 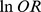 using the simulated
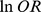
values, thus increasing the reliability of the meta-analysis results.

**Figure 7:**
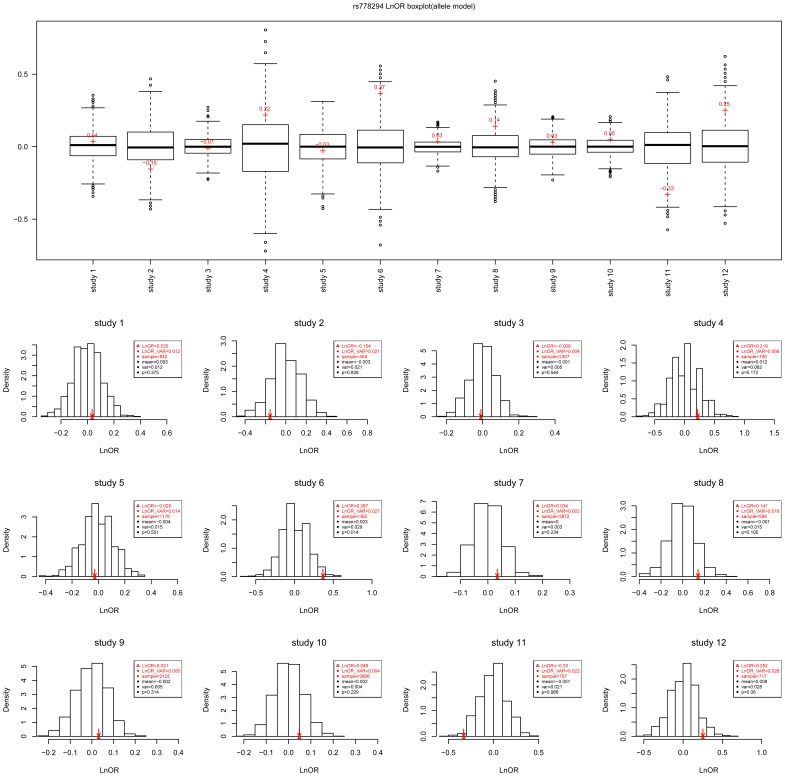
Real LnOR in a random background. (a) Permutation box plot. (b) Permutation probability density.

For each study in a meta-analysis, researchers expect to see the position of a real 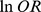
in a random background. To represent this, we designed a permutation box plot as shown for SNP rs778294 in Figure 7a. This allowed us to readily compare the real 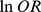 with the random background. In addition, for each study, we plotted the distribution of the random background and the position of the real 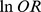
(Figure 7b) to deepen our understanding of the meta-analysis.

### Comparing Running Time between MCPerm and TradPerm

We carried out 1,000 permutations for all 850 SNP loci on a 3.2 GHz desktop PC with a 2G memory running the Windows XP system. TradPerm took about 6 h of computation time, while MCPerm only required 18 min of computation time. This suggests that, for the 850 SNPs examined, MCPerm is about 20 times faster than TradPerm.

We further simulated three genome-wide data sets (100,000, 500,000, and 1,000,000 SNPs) to compare the running time of the two methods. Each data set included 10 genome-wide association studies. We then carried out 1,000 permutations of the three data sets. For 100,000 SNPs, 500,000 SNPs, and 1,000,000 SNPs, MCPerm required approximately 1.5 days, 6–7 days, and 13.5 days of computation time, respectively. The running time was not tested for TradPerm as it would take too long (more than 40 days for 100,000 SNPs).

In addition, for MCPerm, the genotype counts were directly simulated rather than calculated from the shuffled data, so the computation time did not depend on the sample size of a single study. This is directly relevant for a meta-analysis, as the number of studies affects the computation time.

### MCPerm R Package

To facilitate the use of MCPerm, we developed a freely available R package, named MCPerm. The package has detailed instructions and examples and has been uploaded to the Comprehensive R Archive Network (CRAN). Users can download the package at the following website: http://cran.r-project.org/web/packages/MCPerm/index.html. It can be run using both Linux and the Windows environment. The current version is v1.1.4. There are a total of 45 functions that facilitate the implementation of the meta-analysis, TradPerm, and MCPerm. A detailed list of functions is given in Table S1.

## Discussion

Multiple testing is a challenging issue in SNP-based meta-analysis. Among the many multiple testing correction methods, TradPerm is usually considered to be the gold standard [4], [5]. It estimates the background distribution of test *P*-values by shuffling phenotype labels, and thus is an accurate correcting method. However, the over-reliance on raw SNP genotype data and the large amount of computation are two obstacles that limit the scope of TradPerm. To overcome these limitations, we developed a MCPerm method that only uses the summary statistics of genotypes to perform the permutation.

MCPerm simulated the random genotype counts in cases and controls using a two-step hypergeometric distribution. We used five real data sets and five simulation data sets to prove that the genotype count distributions are the same between MCPerm and TradPerm. Furthermore, we showed that MCPerm can obtain the same size of permutation *P*-values of meta-analysis as TradPerm, and take less computation time than TradPerm to do so.

For the meta-analysis of a single SNP locus, we suggest that a MCPerm procedure is carried out as the background distribution of 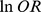
and the position of 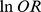
in the background (permutation box plot) will deepen our understanding of the analytical results. In addition, the procedure will enable the variance to be directly calculated from the simulated
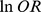
values, increasing the reliability of the meta-analysis.

With the arrival of the era of post-association studies, two-step hypergeometric random numbers in MCPerm may have broader application prospects than expected. In some gene-based and pathway-based association studies, researchers combine *P*-values for SNPs into an overall *P*-value for a gene or pathway [23]. In such studies, the correction of *P*-values for SNPs is essential. However, they are usually undertaken by bioinformatics researchers who are not always able to collect raw SNP genotype data because of privacy policies. The two-step hypergeometric random number in MCPerm will aid these researchers to complete their permutation corrections using only the summary statistics of the genotype data such as those provided by the Wellcome Trust Case Control Consortium [24]. In conclusion, we hope that MCPerm will be widely used in genome-wide association studies and genome-wide studies of meta-analysis.

## Supporting Information

Figure S1 **Cumulative distribution curve of six genotype counts.**
(JPG)

Figure S2 **Comparison of the distributions of the allele model (** ***A*** **allele vs.** ***a*** **allele).**
(JPG)

Figure S3 **Comparison of the distributions of the dominant model (** ***AA+Aa*** **vs.** ***aa*** **genotypes).**
(JPG)

Figure S4 **Comparison of the distributions of the recessive model (** ***AA*** **vs.** ***Aa+aa*** **genotypes).**
(JPG)

Figure S5 **Comparison of meta-analysis** ***P*** **-values of the dominant model.**
(JPG)

Figure S6 **Comparison of meta-analysis** ***P*** **-values of the recessive model.**
(JPG)

Figure S7 **Comparison of heterogeneity test** ***P*** **-values of the allele model.**
(JPG)

Figure S8 **Comparison of heterogeneity test** ***P*** **-values of the dominant model.**
(JPG)

Figure S9 **Comparison of heterogeneity test** ***P*** **-values of the recessive model.**
(JPG)

Figure S10 **Scatter plots of 850 MCPerm heterogeneity test** ***P*** **-values against 850 TradPerm heterogeneity test** ***P*** **-values.** The MCPerm *P*-values are highly consistent with TradPerm *P*-values (*r = *0.999; P<2.2e-16)
(JPG)

Table S1 **The functions in MCPerm R package (v 1.1.4).**
(DOC)

Supporting Information S1 http://www.bioapp.org/research/MCPerm/index.html.
