# Supplementary figures and images for: MCPerm: A Monte Carlo Permutation Method for Accurately Correcting the Multiple Testing in a Meta-Analysis of Genetic Association Studies
Source: PLoS One. 2014 Feb 21;9(2):e89212. doi: 10.1371/journal.pone.0089212 (PMC3931718; doi:10.1371/journal.pone.0089212)

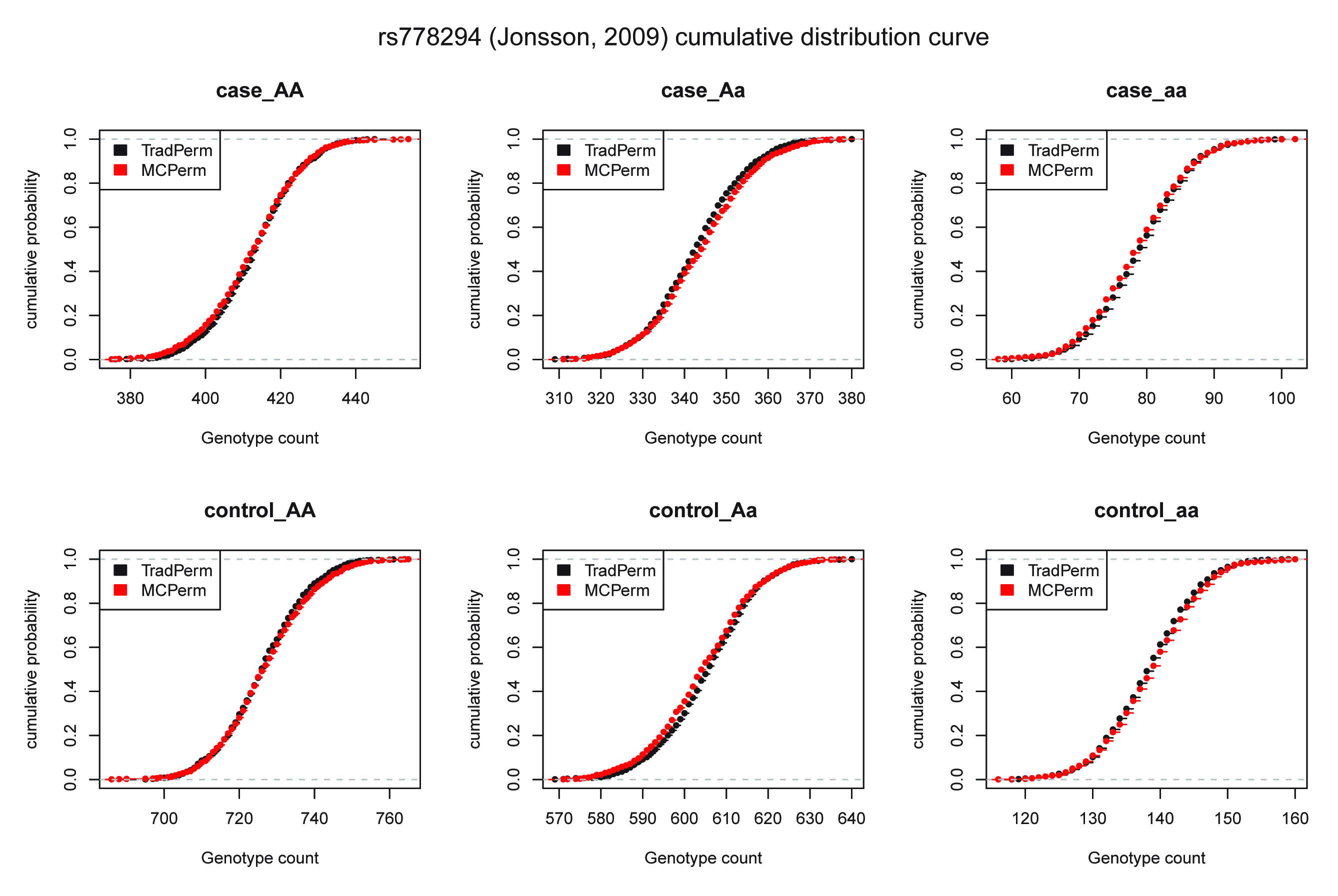

Supplement: Figure S1 — Cumulative distribution curve of six genotype counts. (JPG) [file pone.0089212.s001.jpg]

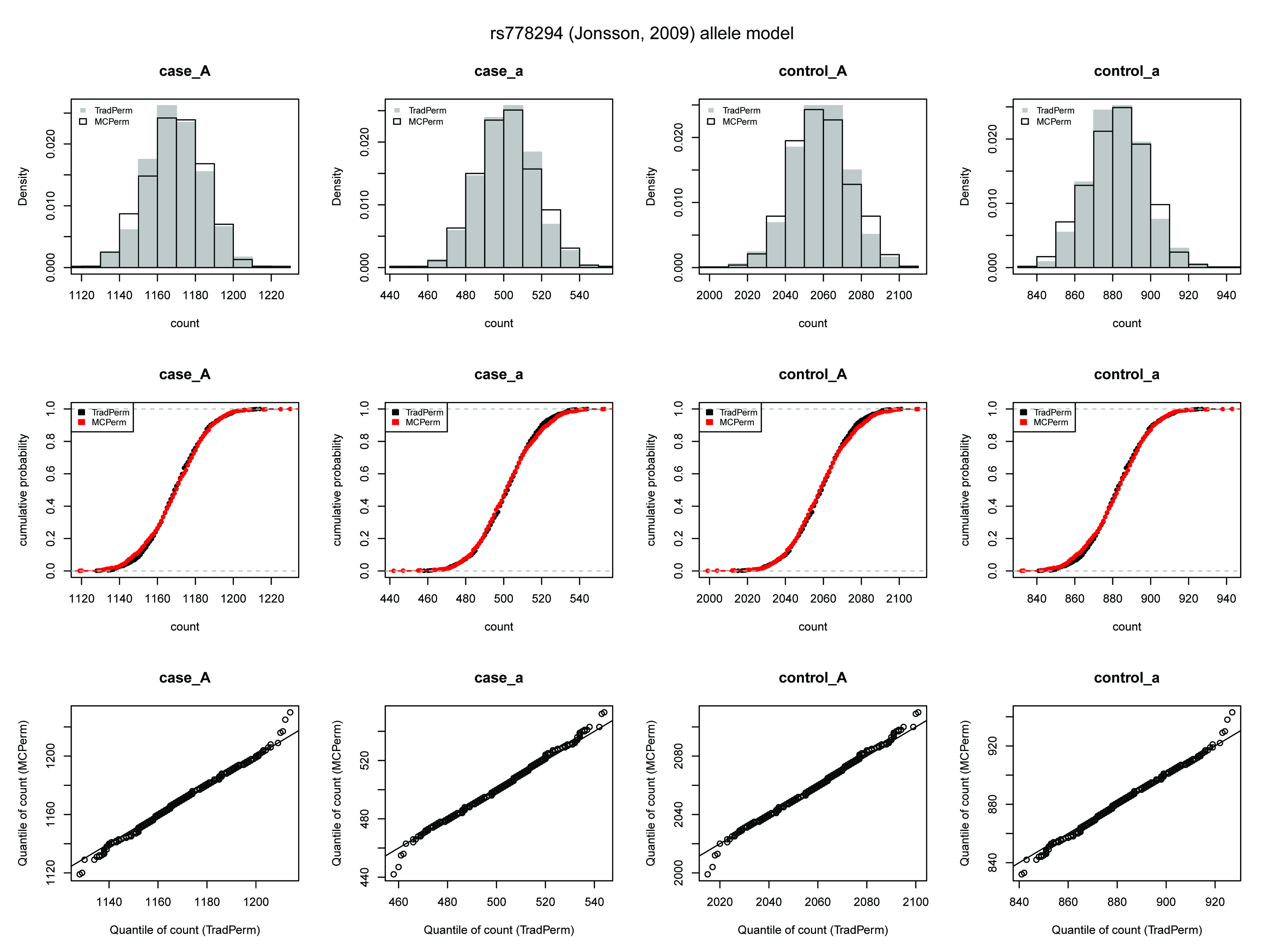

Supplement: Figure S2 — Comparison of the distributions of the allele model ( A allele vs. a allele). (JPG) [file pone.0089212.s002.jpg]

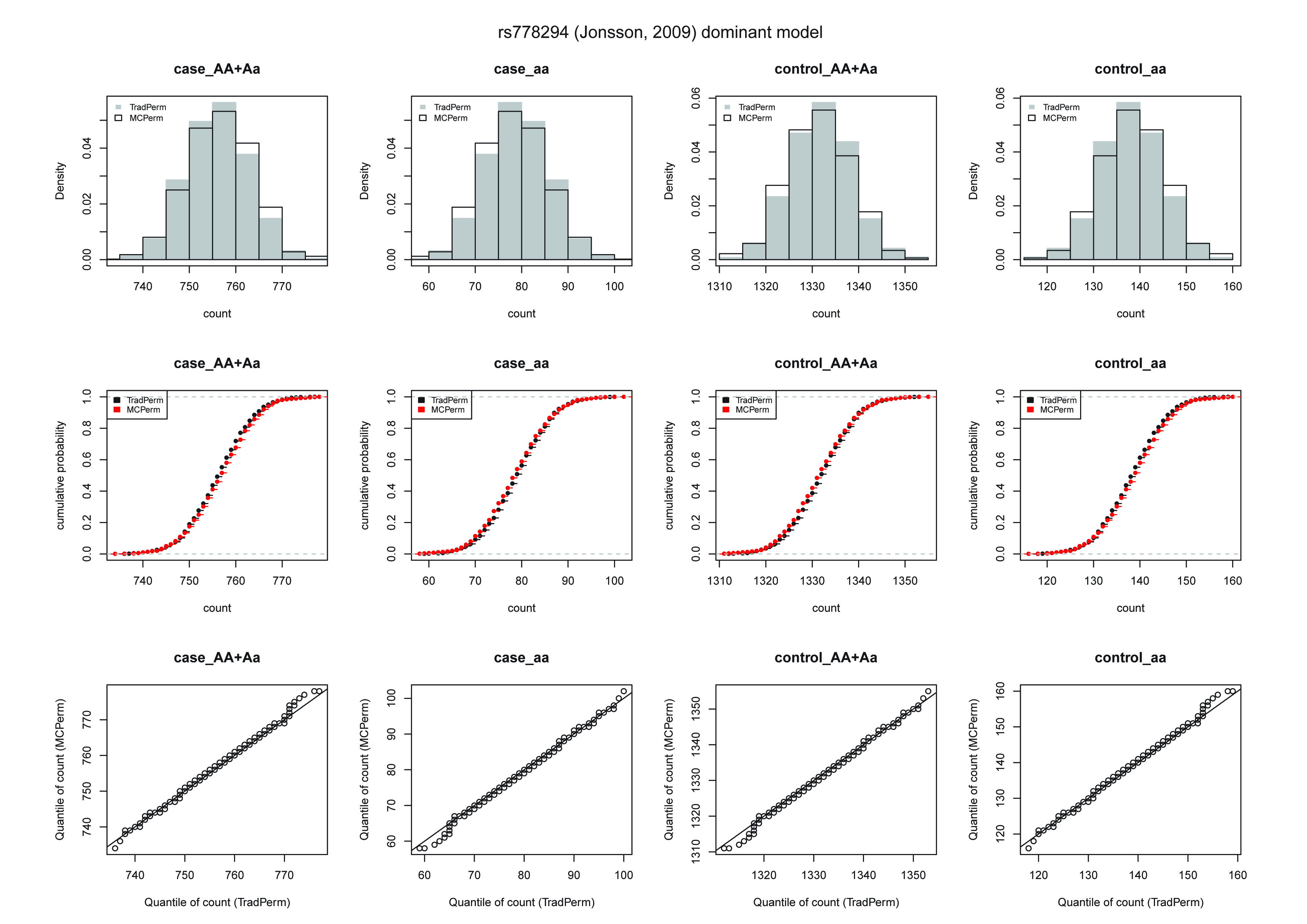

Supplement: Figure S3 — Comparison of the distributions of the dominant model ( AA+Aa vs. aa genotypes). (JPG) [file pone.0089212.s003.jpg]

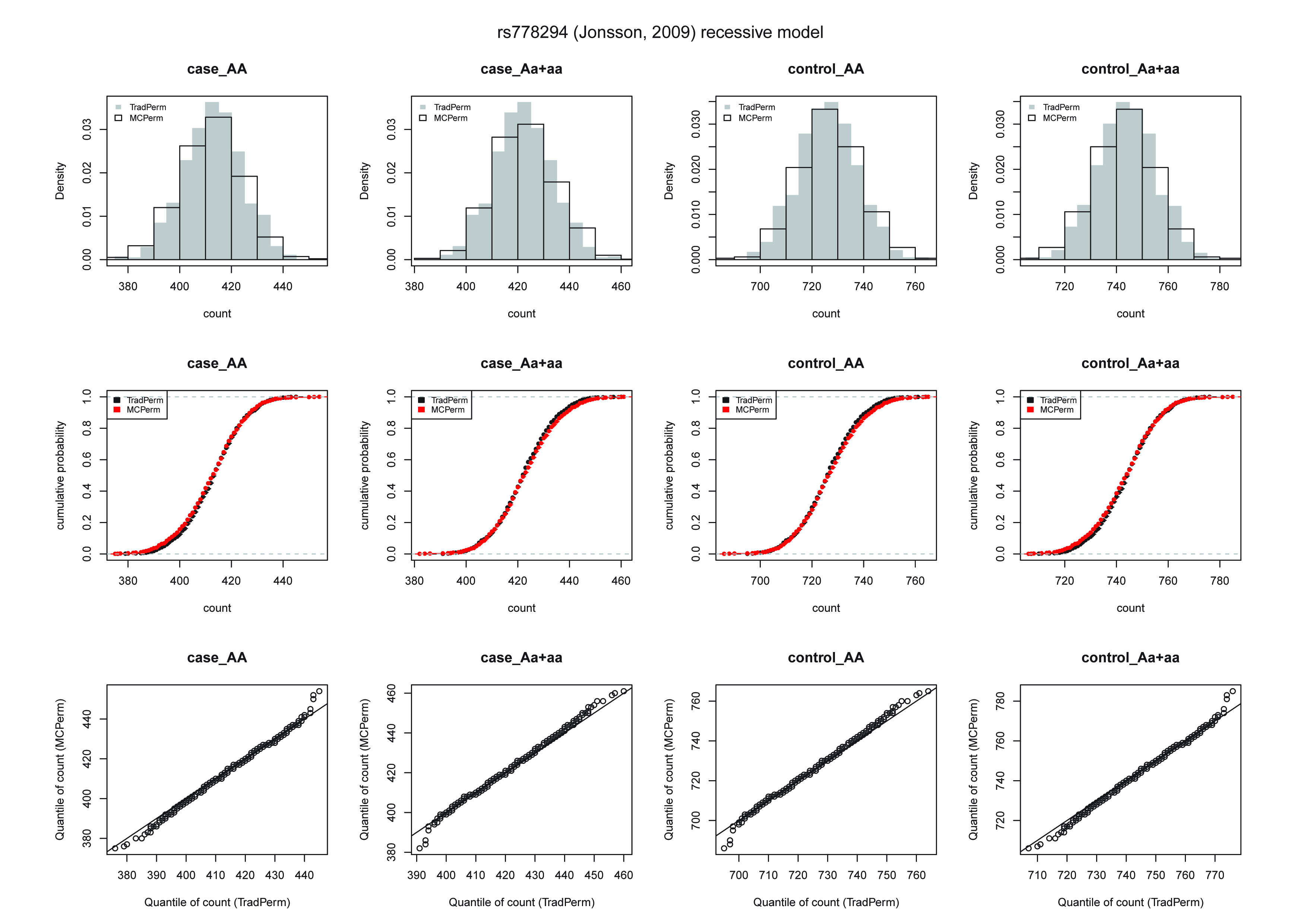

Supplement: Figure S4 — Comparison of the distributions of the recessive model ( AA vs. Aa+aa genotypes). (JPG) [file pone.0089212.s004.jpg]

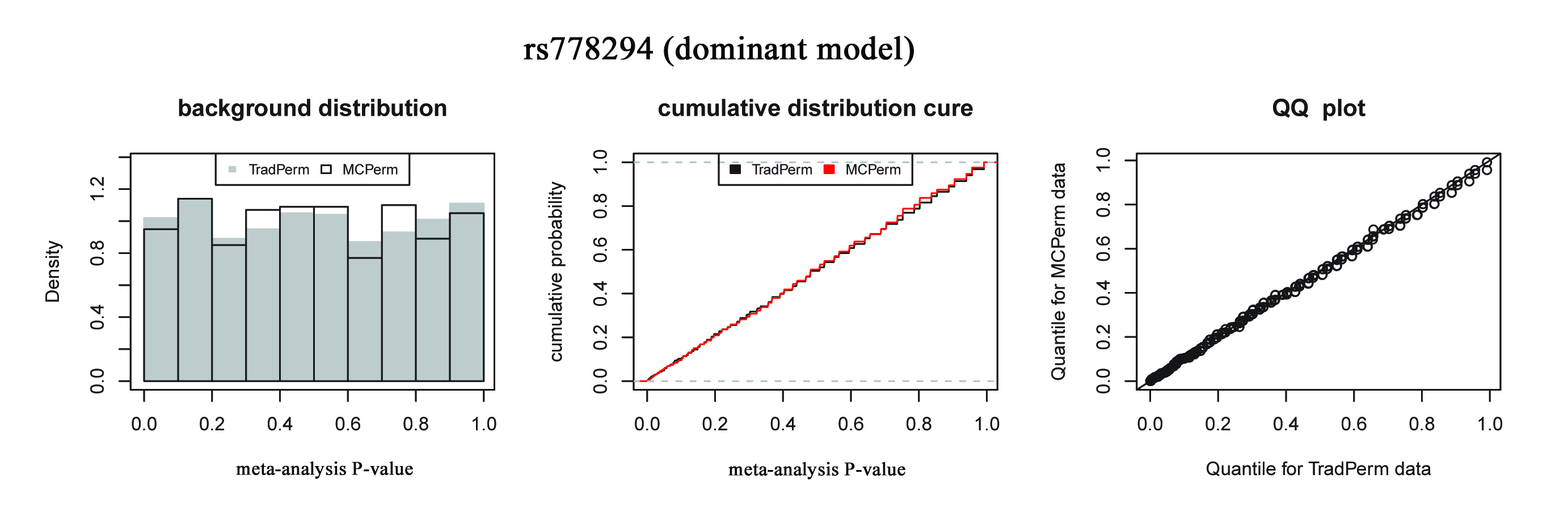

Supplement: Figure S5 — Comparison of meta-analysis P -values of the dominant model. (JPG) [file pone.0089212.s005.jpg]

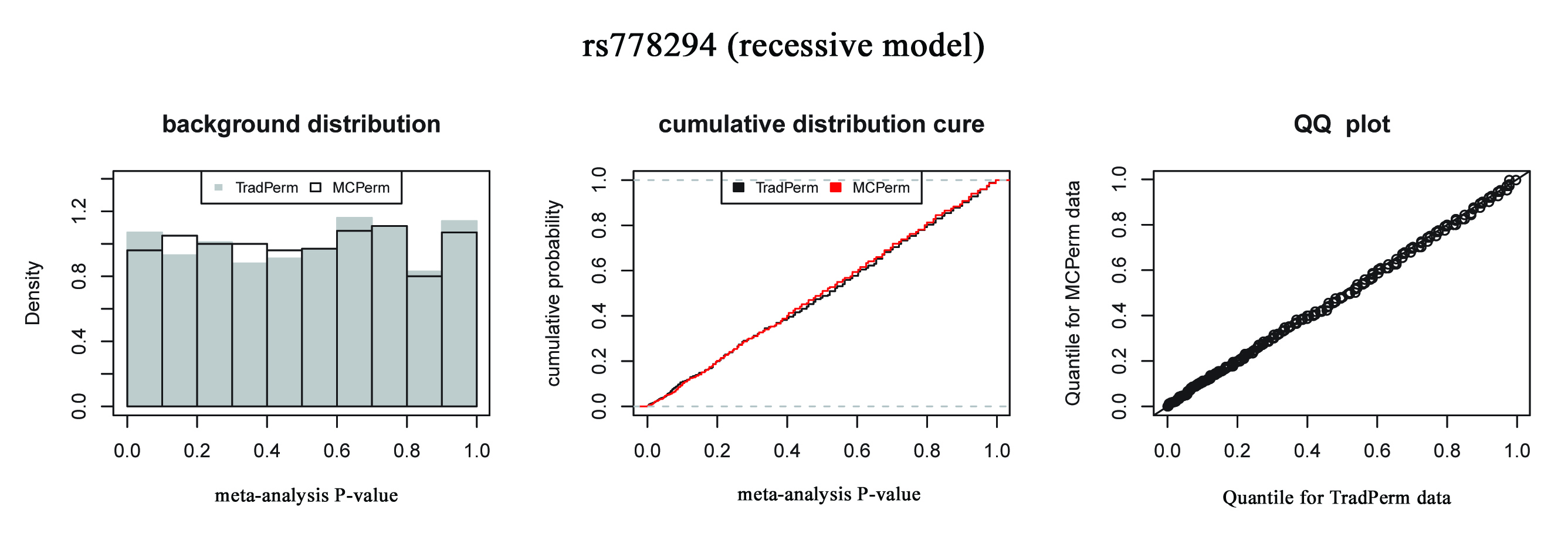

Supplement: Figure S6 — Comparison of meta-analysis P -values of the recessive model. (JPG) [file pone.0089212.s006.jpg]

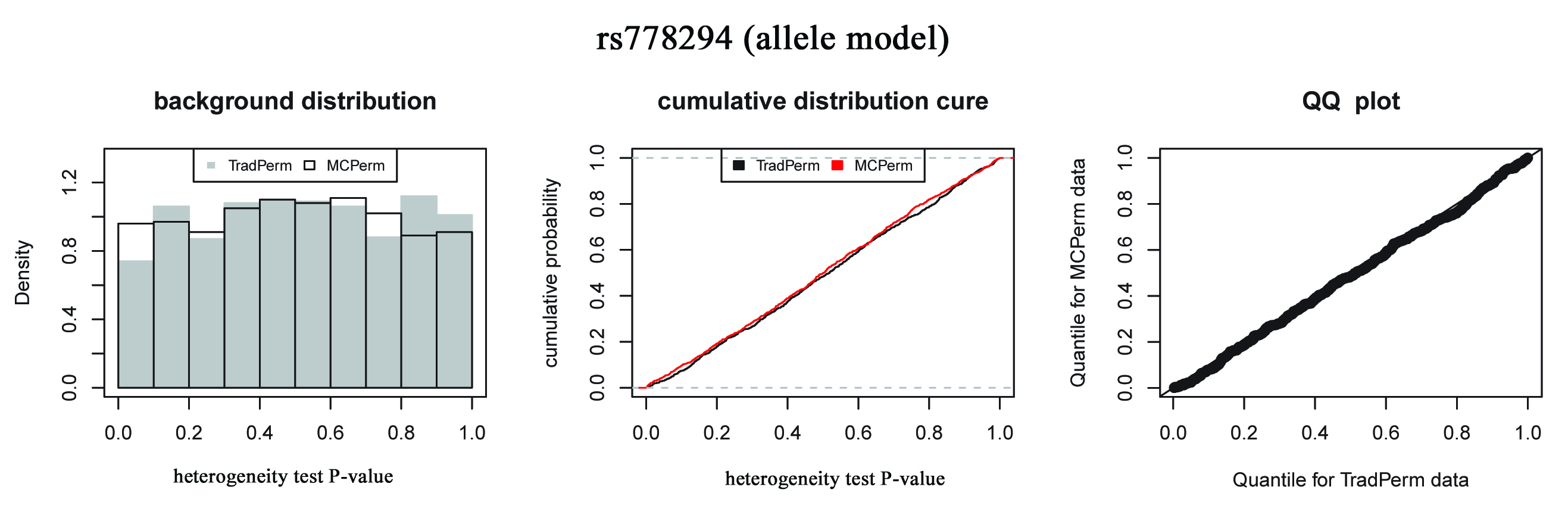

Supplement: Figure S7 — Comparison of heterogeneity test P -values of the allele model. (JPG) [file pone.0089212.s007.jpg]

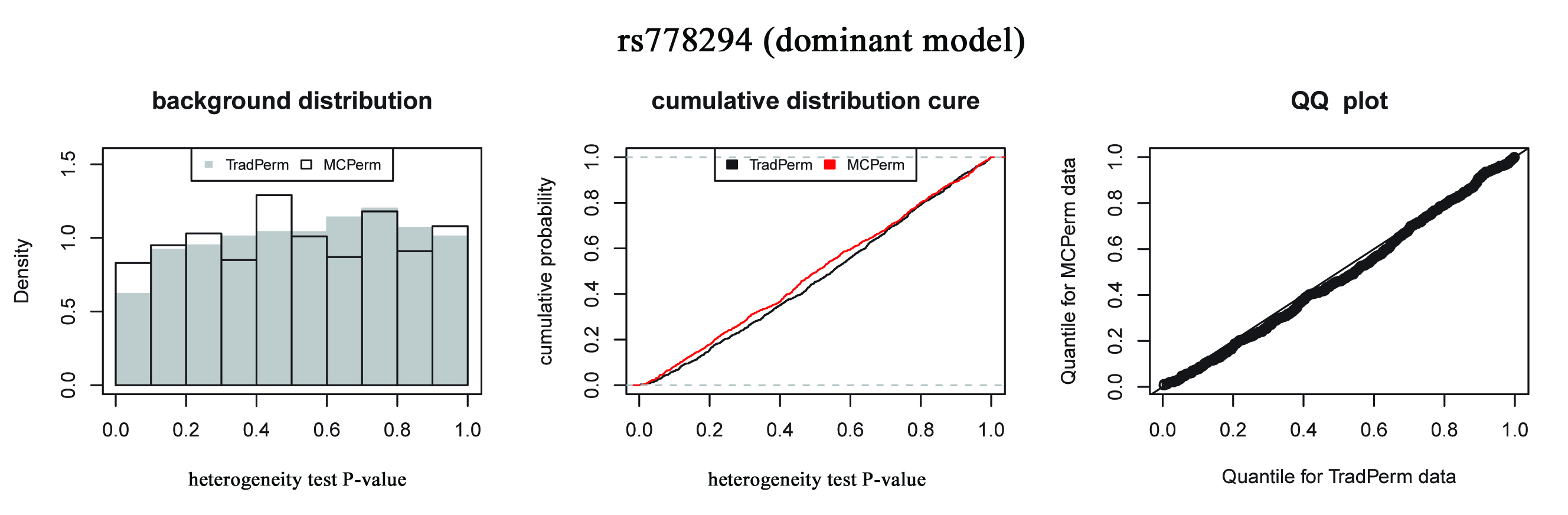

Supplement: Figure S8 — Comparison of heterogeneity test P -values of the dominant model. (JPG) [file pone.0089212.s008.jpg]

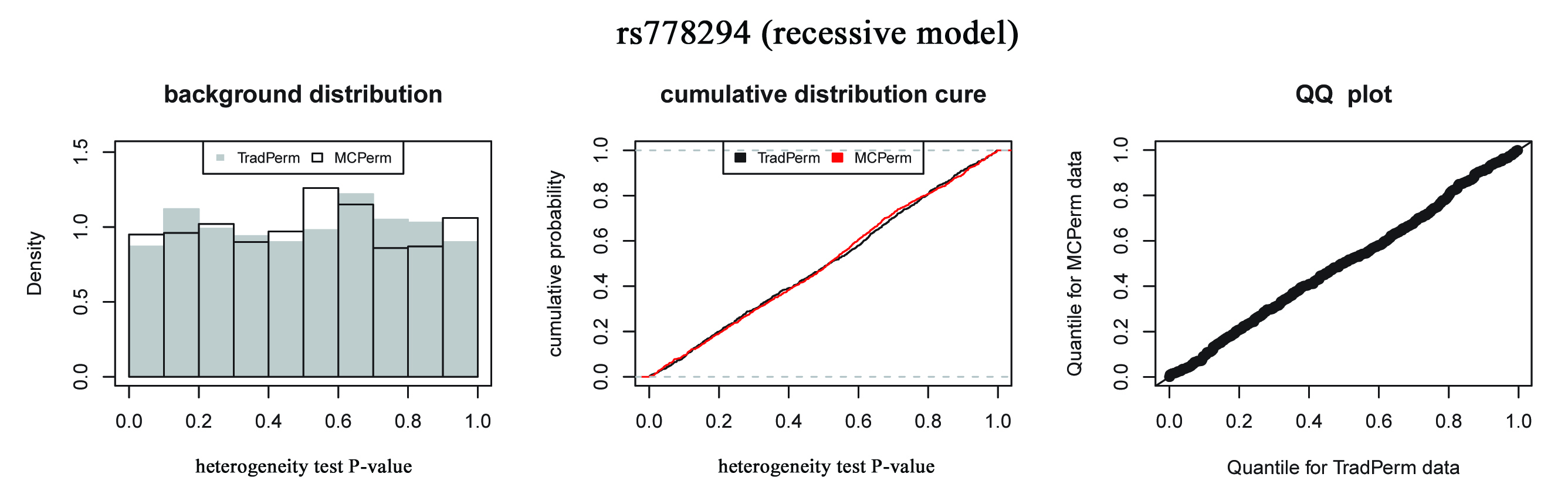

Supplement: Figure S9 — Comparison of heterogeneity test P -values of the recessive model. (JPG) [file pone.0089212.s009.jpg]

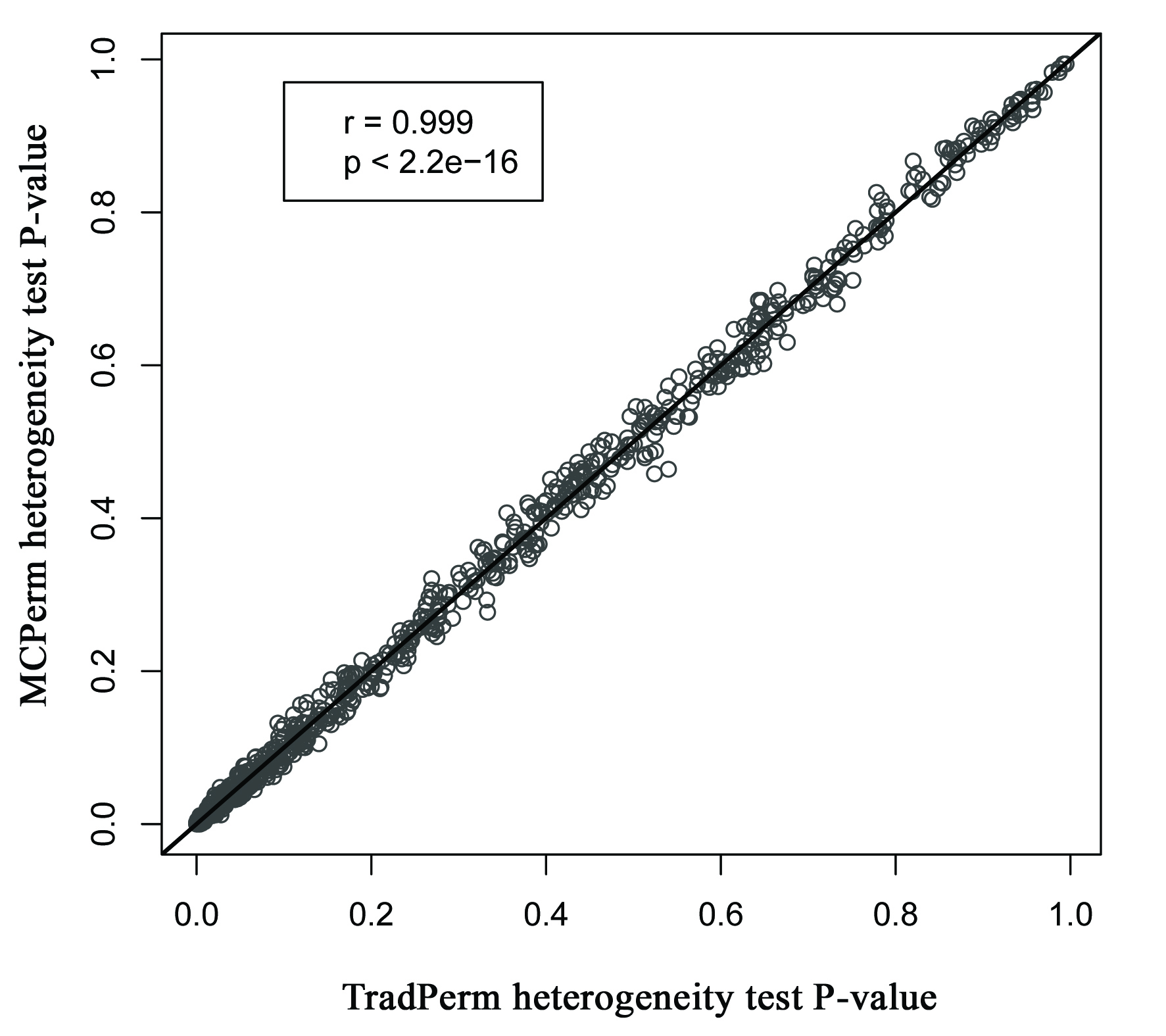

Supplement: Figure S10 — Scatter plots of 850 MCPerm heterogeneity test P -values against 850 TradPerm heterogeneity test P -values. The MCPerm P-values are highly consistent with TradPerm P-values (r = 0.999; P<2.2e-16) (JPG) [file pone.0089212.s010.jpg]
