# Supplementary material for: MCPerm: A Monte Carlo Permutation Method for Accurately Correcting the Multiple Testing in a Meta-Analysis of Genetic Association Studies
Source: PLoS One. 2014 Feb 21;9(2):e89212. doi: 10.1371/journal.pone.0089212 (PMC3931718; doi:10.1371/journal.pone.0089212)
Supplement: Table S1 — The functions in MCPerm R package (v 1.1.4). (DOC) [file pone.0089212.s011.doc]

**Table S1**: the functions in MCPerm R package (v 1.1.4).

| **Function** | **description** |
| --- | --- |
| Armitage | Armitage's trend test for the 2x3 genotype table |
| Armitage.MCPerm | A Monte Carlo permutation method for Armitage's trend test in case/control association study |
| Armitage.TradPerm | A permutation method for Armitage's trend test in case/control association study |
| chisq.MCPerm | A Monte Carlo permutation method for multiple chisq.test correction in case/control association study |
| chisq.TradPerm | A permutation test for multiple chisq.test correction in case/control association study |
| fisher.MCPerm | A Monte Carlo permutation method for multiple fisher.test correction in case/control association study |
| fisher.TradPerm | A permutation test for multiple fisher.test correction in case/control association study |
| genotypeData | Genotype Data from GWA16 |
| genotypeStat | Statistical Allele and Genotype Frequency of the specified snp |
| HW.test | Hardy-weinberg equilibrium test |
| I2.MCPerm | Calculate p.value for Heterogeneity statistics I2 in meta analysis |
| I2.TradPerm | Calculate p.value for Heterogeneity statistics I2 in meta analysis |
| MCPerm | A Monte Carlo permutation method for multiple test correction. |
| meta | Meta analysis |
| meta.MCPerm | Meta analysis corrected by permutation test |
| meta.TradPerm | Meta analysis corrected by permutation test |
| MetaGenotypeCount | rs3131296 genetic association studies from SZGene database |
| MetaGenotypeData | genotype raw data for rs3131296 genetic association studies |
| OR | OR(odd ratio) for risk_allele |
| OR.MCPerm | A Monte Carlo permutation method for multiple OR(odd ratio) test correction in case/control association study |
| OR.TradPerm | A permutation test for multiple OR(odd ratio) test correction in case/control association study |
| pearson_scatter | scatter plot and calculate Pearson correlation coefficient for paired data |
| PermMeta.boxplot | boxplot for the result of 'meta.MCPerm' or 'meta.TradPerm' |
| PermMeta.Hist | histplot for the result of 'meta.MCPerm' or 'meta.TradPerm' |
| PermMeta.LnOR.boxplot | boxplot for the return value 'perm_LnOR' or 'perm_VARLnOR' of 'meta.MCPerm' or 'meta.TradPerm' |
| PermMeta.LnOR.CDC | cumulative distribution curve for the return value 'perm_LnOR' of 'meta.MCPerm' or 'meta.TradPerm' |
| PermMeta.LnOR.Hist | histplot for the return value 'perm_LnOR' or 'perm_VARLnOR' of 'meta.MCPerm' or 'meta.TradPerm' |
| PermMeta.LnOR.qqnorm | qqnorm plot for the return value 'perm_LnOR' of 'meta.MCPerm' or 'meta.TradPerm' |
| permuteGenotype | Permute the elements of genotype data |
| permuteGenotypeCount | Fill the numerics of 2*3 table when fixed the row and column totals |
| print.PermMeta | Print style for function 'meta.MCPerm' or 'meta.TradPerm' |
| Q.MCPerm | Correct p.value for Heterogeneity statistics Q in meta analysis by MCPerm method. |
| Q.TradPerm | Correct p.value for Heterogeneity statistics Q in meta analysis by TradPerm method |
| VS.Allele.CDC | separately plot cumulative distribution curve for the return value(allele count) of 'meta.TradPerm' and 'meta.MCPerm' for certain study |
| VS.Allele.Hist | separately plot histplot for the return value(allele count) of 'meta.TradPerm' and 'meta.MCPerm' for certain study |
| VS.Allele.QQ | separately plot quantile-quantile plot for the return value(allele count) of 'meta.TradPerm' and 'meta.MCPerm' for certain study |
| VS.CDC | plot cumulative distribution curve for the return value of 'meta.TradPerm' and 'meta.MCPerm' for certain study or meta analysis |
| VS.Genotype.CDC | separately plot cumulative distribution curve for the return value(genotype count) of 'meta.TradPerm' and 'meta.MCPerm' for certain study |
| VS.Genotype.Hist | separately plot histplot for the return value(genotype count) of 'meta.TradPerm' and 'meta.MCPerm' for certain study |
| VS.Genotype.QQ | separately plot quantile-quantile plot for the return value(genotype count) of 'meta.TradPerm' and 'meta.MCPerm' for certain study |
| VS.Hist | plot histplot for the return value of 'meta.TradPerm' and 'meta.MCPerm' for certain study or meta analysis |
| VS.KS | Kolmogorov-Smirnov test for the return value of 'meta.TradPerm' and 'meta.MCPerm' |
| VS.QQ | plot quantile-quantile plot for the return value of 'meta.TradPerm' and 'meta.MCPerm' for certain study or meta analysis. |
| MCPerm-package | A Monte Carlo permutation method for multiple test correction. |
| Armitage | Armitage's trend test for the 2x4 genotype table |
