# Supplementary material for: MCPerm: A Monte Carlo Permutation Method for Accurately Correcting the Multiple Testing in a Meta-Analysis of Genetic Association Studies
Source: PLoS One. 2014 Feb 21;9(2):e89212. doi: 10.1371/journal.pone.0089212 (PMC3931718; doi:10.1371/journal.pone.0089212)
Supplement: Supporting Information S1 — http://www.bioapp.org/research/MCPerm/index.html. [file pone.0089212.s012.html]

**Supplementary file 1.**  
Comparison results of 850 loci between MCPerm and TradPerm (meta-analysis P-values and heterogeneity test P-values of allele model).   
**clear here download:**Supplementary file 1.zip(zip file, include 850 .pdf files).
